# Molecular characterization of highly pathogenic H5N1 avian influenza viruses isolated in Sweden in 2006

**DOI:** 10.1186/1743-422X-5-113

**Published:** 2008-10-06

**Authors:** István Kiss, Péter Gyarmati, Siamak Zohari, Karin Wilbe Ramsay, Giorgi Metreveli, Elisabeth Weiss, Maria Brytting, Marielle Stivers, Sofia Lindström, Ake Lundkvist, Kirill Nemirov, Peter Thorén, Mikael Berg, György Czifra, Sándor Belák

**Affiliations:** 1 Joint Research and Development Division in Virology of the National Veterinary Institute (SVA) and Swedish University of Agricultural Sciences (SLU), and Department of Biomedical Sciences and Public Health, Section of Parasitology and Virology, SLU, Ulls väg 2B, SE-751 89 Uppsala, Sweden; 2 Department of Microbiology, Central Agricultural Office, Veterinary Diagnostic Directorate, Bornemissza u. 3-7, H-4031 Debrecen, Hungary; 3 University of Applied Sciences of Weihenstephan, Alte Akademie 1, D-85350 Freising-Weihenstephan, Germany; 4 Swedish Institute for Infectious Disease Control, SE-171 82 Stockholm, Sweden; 5 Molecular Diagnostic Section, Unit for Virology, Immunology, and Parasitology, SVA, Ulls väg 2B, SE-751 89 Uppsala, Sweden

## Abstract

**Background:**

The analysis of the nonstructural (NS) gene of the highly pathogenic (HP) H5N1 avian influenza viruses (AIV) isolated in Sweden early 2006 indicated the co-circulation of two sub-lineages of these viruses at that time. In order to complete the information on their genetic features and relation to other HP H5N1 AIVs the seven additional genes of twelve Swedish isolates were amplified in full length, sequenced, and characterized.

**Results:**

The presence of two sub-lineages of HP H5N1 AIVs in Sweden in 2006 was further confirmed by the phylogenetic analysis of approximately the 95% of the genome of twelve isolates that were selected on the base of differences in geographic location, timing and animal species of origin. Ten of the analyzed viruses belonged to sub-clade 2.2.2. and grouped together with German and Danish isolates, while two 2.2.1. sub-clade viruses formed a cluster with isolates of Egyptian, Italian, Slovenian, and Nigerian origin. The revealed amino acid differences between the two sub-groups of Swedish viruses affected the predicted antigenicity of the surface glycoproteins, haemagglutinin and neuraminidase, rather than the nucleoprotein, polymerase basic protein 2, and polymerase acidic protein, the main targets of the cellular immune responses. The distinctive characteristics between members of the two subgroups were identified and described.

**Conclusion:**

The comprehensive genetic characterization of HP H5N1 AIVs isolated in Sweden during the spring of 2006 is reported. Our data support previous findings on the coincidental spread of multiple sub-lineage H5N1 HPAIVs via migrating aquatic birds to large distance from their origin. The detection of 2.2.1. sub-clade viruses in Sweden adds further data regarding their spread in the North of Europe in 2006. The close genetic relationship of Swedish isolates sub-clade 2.2.2. to the contemporary German and Danish isolates supports the proposition of the introduction and spread of a single variant of 2.2.2. sub-clade H5N1 avian influenza viruses in the Baltic region. The presented findings underline the importance of whole genome analysis.

## Background

The first reports of outbreaks caused by highly pathogenic avian influenza viruses (HPAIV) of H5N1 subtype in 1996 originated from southern China [1]. Systematic influenza surveillances showed that distinct genetic sub-lineages of H5N1 HPAIVs, reflecting on their geographic origin, have been established since then among domestic poultry and have been transmitted to long distances by migratory waterfowl [2,3]. Europe experienced a peak of outbreaks of H5N1 HPAI in domestic poultry and wild birds in March 2006 – that was supposedly the consequence of an unusual westward movement of waterfowl from the Black Sea area [4-6]. The recent avian influenza virus strains of European-Middle Eastern-African (EMA) origin were assigned to three clades (EMA-1-3) based on the phylogeny of the complete genomes of the isolates [7], which are referred as sub-clades 2.2.1.-2.2.3. according to the more recent nomenclature [8]. Further, clade 2.2. was classified into three sub-clades: Clade 2.2.1. appeared in Egypt, southern Germany, Italy, Mongolia, and some regions in sub-Sahara Africa. Clade 2.2.2. viruses were detected in northern Germany, Denmark, Sweden, Scotland, and Nigeria, while clade 2.2.3. viruses were demonstrated in India, Afghanistan, Italy, and Iran [9]. Simultaneous transmission of different strains was reported in several European countries such as Sweden [10], Germany [9], France and Italy [11]. Characterization of the Swedish H5N1 HPAIV isolates based on the nonstructural (NS) gene nucleotide sequences demonstrated that all belonged to clade 2.2. The majority of them clustered together with clade 2.2.2., viruses belonging to clade 2.2.1. were also introduced into Sweden [10].

The aim of this study was to further investigate the Swedish H5N1 HPAI viruses by sequencing twelve selected isolates representing four east-coast provinces of the area affected by the epidemic during March-April 2006. The sequence information was used to study the evolution and epidemiology of the outbreak of H5N1 in Europe during 2006. Further, a H5N1 strain isolated from a mink was investigated to reveal any possible adaptation towards mammals.

## Results and discussion

### Phylogenetic analysis

According to the Influenza A Virus Genotype Tool [12] the studied genes of the investigated Swedish isolates belonged to the following lineages: PB2 (K), PB1 (G), PA (D), HA (5J), NP (F), NA (1J), MP (F), NS (1E).

**Figure 1:**
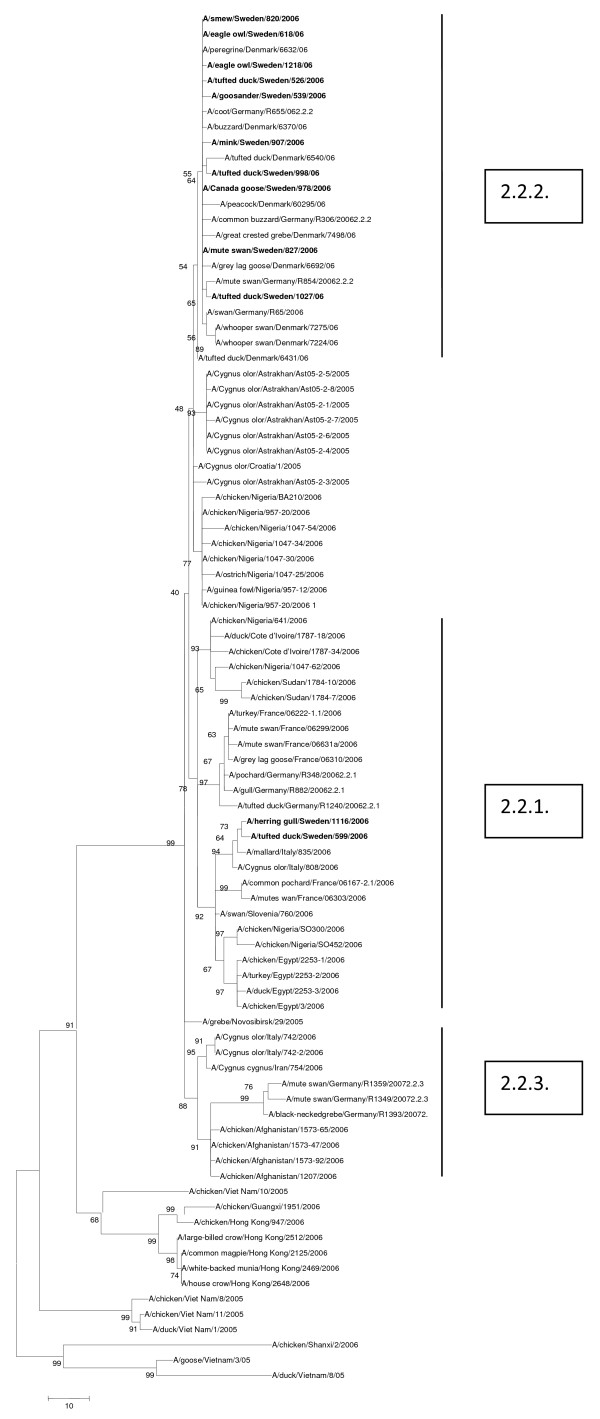
**Evolutionary relationships of HA genes of Swedish HP H5N1 AIVs compared to genetically closely related H5N1 viruses isolated in Europe.** The phylogenetic trees were generated by maximum parsimony analysis (neighbor-joining revealed similar tree topologies). Bootstrap values of 1000 resamplings in per cent are indicated at key nodes. The Swedish viruses are highlighted by bold letters.

**Figure 2:**
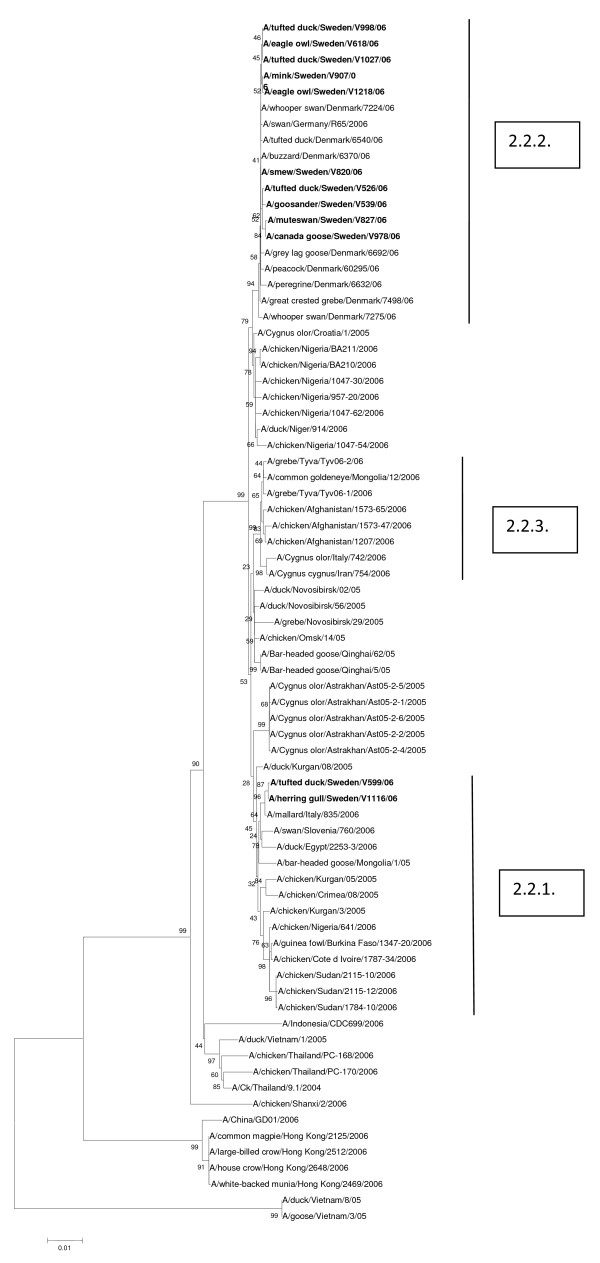
**Evolutionary relationships of PB2 genes of Swedish HP H5N1 AIVs compared to genetically closely related H5N1 viruses isolated in Europe. **The phylogenetic trees were generated by maximum parsimony analysis (neighbor-joining revealed similar tree topologies). Bootstrap values of 1000 resamplings in per cent are indicated at key nodes. The Swedish viruses are highlighted by bold letters.

All twelve Swedish H5N1 isolates in this study belonged to the 2.2. clade and the phylogenetic trees of all eight genes had similar topologies. Representative trees of the HA and PB2 genes are shown (Figures 1 and 2). These data along with those generated from the other genes confirmed the close genetic relationship of H5N1 HPAIVs isolated in the northern region of Germany, Denmark and Sweden in early 2006. Two isolates out of the Swedish ones (A/tufted duck/Sweden/599/06 and A/herring gull/Sweden/1116/06) grouped together with sub-clade 2.2.1. viruses while the other ten belonged to sub-clade 2.2.2. No viruses of sub-clade 2.2.3. were identified among the studied ones.

HA amino acid residue 403 was observed to characterize 2.2.1. (isolates mainly from Southern parts of Germany) and 2.2.2. (German isolates from the North) sub-clade German H5N1 viruses because the former group contained mainly D while the latter N at this position. All sub-clade 2.2.2. Swedish H5N1 viruses possessed N at HA 403 position together with A/tufted duck/Sweden/599/06 sub-clade 2.2.1. isolate, and only 2.2.1. isolate A/herring gull/Sweden/1116/06 had D at this site.

As far as the NA gene concerned residues 34 I/V, 44 C/R, 305 S/N appeared to be discriminative of sub-clade 2.2.1./2.2.2. isolates, respectively, consistently in case of Swedish viruses and predominantly in the analyzed additional 100 sequences. Also, at NA amino acid position 305 sub-clade 2.2.1. Swedish isolates uniquely had an S while all other viruses that were analyzed (sub-clade 2.2.2. and 2.2.3. viruses) possessed N at this position due to a A*A*T→A*G*T transition. Sub-clade 2.2.2. Swedish viruses, and A/great crested grebe/Denmark/7498/06, A/grey lag goose/Denmark/6692/06, A/buzzard/Denmark/6370/06, A/tufted duck/Denmark/6540/06, and A/swan/Germany/R65/06 isolates possessed D at NA position 316 while all other analyzed viruses had G at this site.

The separation of the Swedish H5N1 HPAIVs into two subgroups was already demonstrated on the basis of NS gene sequences [10] and this finding was consistent for all eight genes of the isolates (herein summarized in Additional file 1). No reassortant variant was found among the sequenced twelve Swedish isolates.

### Molecular characterization

Characteristic findings regarding the preservation/substitutions at particular amino acid positions along with potential distinctive molecular markers for the Swedish H5N1 viruses are summarized in Additional file 1.

### Polymerase genes

A single amino acid substitution, from glutamic acid (E) to Lysine (K) in position 627 in PB2 is a determinant of mammalian host range [13,14]. Most avian isolates have E in this position. The substitution to K in this position converts a nonlethal H5N1 influenza A virus isolated from a human to a lethal virus in mice [13]. Among the H5N1 HPAIV sequences we investigated a larger proportion of those originating from 1998–2005 had PB2-E627 than more recent isolates. The 2.2.2.-like Swedish viruses along with the most closely related Danish and German isolates encoded K at this site while the two sub-clade 2.2.1.-like Swedish isolates (A/tufted duck/Sweden/599/06 and A/herring gull/Sweden/1116/06) possessed E at position 627. The mutations D701N and S714R in PB2 contribute to virulence by enhancing polymerase activity [15]. All Swedish isolates had D and S at position 701 and 714, respectively.

PB1-F2 has been identified as a proapoptotic mitochondrial protein expressed from a second open reading frame of the PB1 gene [16] and it has been shown to contribute to viral pathogenesis in mice [17]. Aspargine in position 66 in PB1-F2 has been demonstrated to play a key role in the pathogenicity of H5N1 viruses [18] and its presence was determined in all Swedish viruses. Furthermore, Swedish 2.2.1. subclade viruses had a K26Q substitution compared to 2.2.2. subclade viruses. Isolate A/tufted duck/Sweden/599/06 solely contained a T323I and a H562P, while A/herring gull/Sweden/1116/06 a V719M substitution, respectively. The H5N1 viral polymerase activity is enhanced by the presence of PB2 701N and 714R, PB1 13P, PA 615N, further, NP 319K and 678N [15]. Among the Swedish isolates the presence of PB1 13P was determined.

### Surface glycoprotein genes

The HA sequences of isolates A/Mute swan/Sweden/827/06, A/Canada goose/Sweden/978/06, and A/peregrine/Denmark/6632/06 proved to be identical. The amino acid sequence flanking the cleavage site of the HA gene was PQGERRRKKRGLF alike all other 2.2. viruses with the exception of some French isolates that had the PQGERKRKKR/G sequence [11]. The identified amino acid markers of H5N1 influenza viruses isolated at Qinghai and Poyang Lakes from migratory birds (HA-I99, HA-N268, and NA-R110) were present in all Swedish isolates as well [11]. No "sub-clade"-specific amino acid changes were identified in the HA among the two subgroups of Swedish isolates. All the Swedish isolates had the 238Q and 240G (numbered from the H5 start codon) which indicates preferred receptor specificity for the avian α-(2,3) linkage to galactose [19,20]. All HA sequences contained 6 N-linked potential glycosylation sites, as analysed with NetNglyc server (threshold: 0.5) at the following positions: 27, 39, 181, 302, 500, 559; none of them is adjacent to the cleavage site. Furthermore, the substitutions S145L and A172T, which are associated with viral adaptation to poultry [21] were not determined in association with the Swedish H5N1 viruses.

The amino acid substitutions R178I and I248V in HA that were found in the domestic birds of the Danish isolates [22] were not present in any of the Swedish viruses, nor the V73I substitution that was found in the Danish swan isolates. However, the D387N substitution found in the German and most of the Danish isolates was also present in the Swedish isolates.

The H5N1 virus isolated from a mink (A/Sweden/mink/2006/V907) was examined in order to reveal any possible adaptation towards mammals. As a result, a unique E513G substitution was found in the HA gene but no substitutions that could be regarded as host-related were found, which is consistent with previous findings, i.e. that a single passage in mammals is not necessarily associated with changes in receptor-binding sites [9].

As in the other 2.2. viruses, NA-R110 was present in the Swedish isolates, and a 20 amino acid deletion was also found at positions 49–68 similarly to the majority of the recent H5N1 strains [22]. The N228S substitution was present only in A/Herring gull/1116/06 Swedish 2.2.1. virus (alike with several other member of the sub-clade) and not in A/Tufted duck/Sweden/599/06 isolate. These two isolates differed further in amino acid residues 414 and 434 by bearing N/K and S/G corresponding to A/Herring gull/1116/06 and A/Tufted duck/Sweden/599/06 viruses, respectively. Interestingly, while the Danish and German isolates shared unique amino acids in the NA (G336D), PB1 (K531R) and NS2 (G63E) proteins the Swedish isolates were not homogenous in this regard: although NA-G336D was a characteristic of the Swedish viruses too, two isolates retained the PB1-531K, and NS2-63G. Reported substitutions in NA, inducing oseltamivir resistance [9], were not found in the Swedish isolates.

### The NP and M genes

The NP-10Y amino acid residue, which may affect the pathogenicity of AIVs [15], was present in all of the Swedish isolates. Concerning the M2 gene, all Swedish viruses contained the L26-V27-A30-S31-G34 amino acid pattern, thus, no adamantan drug resistant variant was revealed [9]. Substitutions S64A and E66A that were present in the M2 genes of H5N1 AIV isolates from Hong Kong [11] did not appear in Swedish viruses.

The complete characterization of the NS genes from these isolates was described by Zohari et al., [10], and is not further discussed here.

The effect of substitutions on the predicted antigenicity was investigated among the Swedish isolates for the surface glycoproteins (HA and NA) and for those primarily targeted by the host's cellular immune response (PB2, PA, and NP [23]) (Table 1). The observed amino acid alterations affected the predicted antigenic epitopes in few cases. Regarding the HA in all but one cases 22 epitopes were predicted by the Kolaskar-Tongaonkar approach [24], the exception was strain A/herring gull/Sweden/1116/06, bearing a V201M substitution, which resulted in splitting the corresponding GKLCDLDGVKPLILRDCSVAGW predicted epitope (between amino acid residues 55–76) into two smaller ones: GKLCDLD (aa residues 55–61) and PLILRDCSVAGW (aa residues 65–76). The predicted numbers of epitopes in NA were higher for Swedish 2.2.1. viruses than for 2.2.2. viruses (19–20 compared to 17–18). However, in this case no splitting of epitope(s) was predicted due to a change in the amino acid sequence, but rather, the substitutions could be associated with the appearance of newer epitopes (data not shown). No changes in the number of predicted epitopes was found in for PB2 and PA. In general, the Swedish viruses coded for 15 epitopes on the NP with the only exception of sublineage 2.2.2. virus A/eagle owl/Sweden/V618/06, which had an additional epitope of seven amino acids between residues 22–28. In summary, the detected amino acid changes among the Swedish viruses appeared to have greater effect on the composition of proteins targeted by the humoral than those targeted by the cellular immune responses, in particular, on the NA gene.

**Table 1:** Differences in number of nucleotide and amino acid compositions, synonymous and nonsynonymous nucleotide substitutions, and predicted antigenic epitopes between sub-clade 2.2.1.-2.2.2. Swedish H5N1 avian influenza viruses.

Gene	Region of comparison/nucleotide/	Difference between sub-clade 2.2.1.-2.2.2. Swedish viruses		Number of synonymous/nonsynonymous nucleotide changes		Average number of predicted antigenic epitopes	
		Average number of nucleotide differences	Average number of amino acid differences	2.2.1.	2.2.2.	2.2.1.	2.2.2.

PB2	73–2193	23.7	4.5	0/1	6/4	32	32

PB1	22–2199	20.1	6.9	6/3	10/4	Nd	Nd
		PB1-F2: 1.1	1.1	0/0	0/1		

PA	60–2091	13	3.2	1/0	3/2	28	28

HA	49–1636	15.5	1.5	1/2	5/5	22.5	22

NP	1–1497	16.1	6.2	2/5	8/10	15	15.1

NA	1–1344	14.8	8.6	10/6	9/13	19.5	17.8

MP	MP1: 1–950	12	4.2	3/2	9/14	Nd	Nd
	MP2: 1–262	5.3	2.9	0/2	4/6		

NS	NS1: 1–678	9.3	3.7	1/1	8/14		
	NS2: 1–366	6.8	3.5	1/1	9/11	Nd	Nd

Nd: Not done

## Conclusion

The incursion of H5N1 HPAIV strains falling into three sub-clades into Europe throughout late 2005 and 2007 has been demonstrated earlier [7]. Further reports and the analysis of the corresponding published sequences revealed the introduction of multiple variants of H5N1 HPAIV into several European countries, such as sub-clade 2.2.1. and 2.2.2. viruses into Germany, France, and Sweden [6,9,11,25], and subclade 2.2.1. and 2.2.3. viruses into Italy [7]. The Swedish 2.2.1. sub-clade viruses were closely related to A/Cygnus olor/Italy/808/2006 and A/mallard/Italy/835/2006 and shared several common nucleotide and amino acid motifs, among them, importantly, the PB2-627E, suggesting that they derived from an earlier progenitor of Southeast Asian origin. The detection of these H5N1 HPAIV strains in Sweden adds further data regarding the spread of 2.2.1. viruses in the North. The accumulation of particular mutations reflects that presumably these viruses have been circulating in the South before the transmission to the northern parts of Europe [9]. Sub-clade 2.2.2. Swedish H5N1 HPAIV isolates proved to be closely related to the contemporary German and Danish isolates, which supports the proposition of the introduction and spread of a single variant of 2.2.2. sub-clade H5N1 avian influenza viruses in the Baltic region.

The number and composition of the immune reactive peptides predicted by computing indicated that the surface glycoprotein genes were more affected than the nucleoprotein, polymerase basic protein 2, and polymerase acidic protein, the main targets of the cellular immune responses.

The above observations, alike those with similar objectives, highlight and warrant the importance of whole genome sequencing of HPAIV isolates, in order to improve the surveillance and preparedness against highly pathogenic avian influenza.

## Methods

### Viral isolates

The isolates involved in this study are shown in Table 2. They were collected during the HPAI outbreak in Northern Europe in spring 2006 [10].

**Table 2:** List of the H5N1 HPAIV isolates used in this study

**Isolate name**	**Species**
A/tufted duck/Sweden/V526/06	*Aythya fuligula*

A/goosander/Sweden/V539/06	*Mergus merganser*

A/tufted duck/Sweden/V599/06	*Aythya fuligula*

A/eagle owl/Sweden/V618/06	*Bubo bubo*

A/smew/Sweden/V820/06	*Mergus albellus*

A/mute swan/Sweden/V827/06	*Cygnus olor*

A/mink/Sweden/V907/06	*Mustela vison*

A/canada goose/Sweden/V978/06	*Branta canadensis*

A/tufted duck/Sweden/V998/06	*Aythya fuligula*

A/tufted duck/Sweden/V1027/06	*Aythya fuligula*

A/herring gull/Sweden/V1116/06	*Larus argentatus*

A/eagle owl/Sweden/V1218/06	*Bubo bubo*

For further details of the viruses see reference Zohari et al., 2008 [10].

### RT-PCR and nucleotide sequencing

The collection of specimens, RNA extraction, and RT-PCR amplification of the NS1 sequences was described earlier and the same RNA batches were used for this study that served as targets in the previous investigation [10]. In order to obtain possibly the full length nucleotide sequences of the coding regions of the influenza virus isolates several approaches were combined that comprised of either published protocols/primers [22,26,27] or those developed and used by the Influenza Genome Sequencing Project [[28]; the primer sequences were kindly provided by David Spiro, The J. Craig Venter Institute, Rockville, Maryland, USA), or designed by ourselves. The primer and PCR protocols for sequencing are available from the authors upon request.

### Phylogenetic analysis

For the phylogenetic analyses, a set of H5N1 AIVs that were isolated in Europe, Asia and Africa in 2005 – 2006 was selected and used for all genes. These were collected from the Influenza Virus Resource at NCBI [29] and these were included in the phylogenetic analyses.

Sequence assembly, multiple alignment and alignment trimming were performed with the CLC Combined Workbench 3.0.2. bioinformatics software (CLC bio A/S, Aarhus, Denmark). Distance based neighbor joining and character based maximum parsimony phylogenetic trees were generated using the Molecular Evolutionary Genetics Analysis (MEGA) software v.4.0. [30] with 1000 bootstrap replicates. For the neighbor-joining trees, the Kimura-2 substitution model was used. Other models were also tested which showed similar topologies. The evolutionary divergence between the sub-clades was investigated by pairwise analyses over all sequence pairs between the groups by using the MEGA software also. The occurrence and distribution of synonymous and nonsynonymus substitutions was investigated by the DNA Sequence Polymorphism software (Version 4.50.3) software [31]. Computational analysis of the antigenic sites was carried out by using the Kolaskar-Tongaonkar method [24].

### Nucleotide sequence accession numbers

Nucleotide sequences from Swedish H5N1 virus isolates included in this study have been submitted to GenBank with the following accession numbers: PB2: EU889035–EU889046, PB1: EU889047–EU889058, PA: EU889059–EU889070, HA: EU889071–EU889082, NP: EU889083–EU889094, NA: EU889095–EU889106, M: EU889107–EU889118.

## Competing interests

The authors declare that they have no competing interests.

## Authors' contributions

IK took part in conception and organized protocol developments, performed sequence analyses, alignments, phylogenies, drafted and wrote the manuscript. PG took part in conception, developed amplification protocols, performed sequence analyses, alignments, phylogenies, contributed to and revised the manuscript. SZ propagated the viruses, provided nucleotide sequences and core data, contributed to the interpretation of the findings and to the writing of the manuscript. KWR took part in conception, performed sequence analyses, alignments, phylogenies, contributed to and revised the manuscript. GM carried out a large portion of PCR and sequencing reactions, sequence data analysis, and contributed to the writing of the manuscript. EW optimized the assays initially and run much of the amplification reactions, helped in literature search and data analysis. MBrytting contributed to conception, took part in and organized data analyses, revised the manuscript. MS and SL participated in sequencing and method optimization, and took part in data analysis and interpretation. AL contributed to conception, organized data analyses, and revised the manuscript. KN took part in the PCR runs and sequencing reactions and contributed to the writing of the manuscript. PT, MBerg, and GC contributed to conception, interpretation of data, and revised the manuscript. BS critically revised the manuscript and gave the final approval for publication.

All authors read and approved the final manuscript.

## Supplementary Material

Additional file 1 Main amino acid characteristics of the Swedish H5N1 HPAIV isolates. Some major amino acid residues characterizing and discriminating subclade 2.2.1. and 2.2.2. Swedish H5N1 HPAIV isolates are summarized in the table. Click here for file
