# Supplementary material for: Molecular characterization of highly pathogenic H5N1 avian influenza viruses isolated in Sweden in 2006
Source: Virol J. 2008 Oct 6;5:113. doi: 10.1186/1743-422X-5-113 (PMC2569034; doi:10.1186/1743-422X-5-113)
Supplement: Additional file 1 — Main amino acid characteristics of the Swedish H5N1 HPAIV isolates. Some major amino acid residues characterizing and discriminating subclade 2.2.1. and 2.2.2. Swedish H5N1 HPAIV isolates are summarized in the table. [file 1743-422X-5-113-S1.doc]

**Main amino acid characteristics of the Swedish H5N1 HPAIV isolates.**

|  | PB2 | | | PB1 | | | | | | PA | | | HA | | | | | | | | NP | | | | | | | NA | | | | M2 | | | | | |
| --- | --- | --- | --- | --- | --- | --- | --- | --- | --- | --- | --- | --- | --- | --- | --- | --- | --- | --- | --- | --- | --- | --- | --- | --- | --- | --- | --- | --- | --- | --- | --- | --- | --- | --- | --- | --- | --- |
| Strain/aa residue | 627 | 701 | 714 | 13 | 57 | 182 | 213 | 531 | 654 | | 70 | 615 | | 99 | 145 | 172 | 238 | 240 | 403 | Cleavage site | 10 | | 270 | 313 | 319 | 371 | 34 | | 44 | 305 | 316 | 26 | 27 | 30 | 31 | 34 |  |
| A/Tufted duck/Sweden/V526/06 | K | D | S | P | T | T | N | R | G | | A | K | | I | S | A | Q | G | N | PQGERRRKKRGLF | Y | | I | F | N | M | V | | R | N | D | L | V | A | S | G |  |
| A/Goosander/Sweden/V539/06 | K | D | S | P | T | T | N | R | G | | A | K | | I | S | A | Q | G | N | PQGERRRKKRGLF | Y | | I | F | N | M | V | | R | N | D | L | V | A | S | G |  |
| A/Tufted duck/Sweden/V599/06 | E | D | S | P | K | I | K | K | S | | T | K | | I | S | A | Q | G | N | PQGERRRKKRGLF | Y | | V | S | N | T | I | | C | S | G | L | V | A | S | G |  |
| A/Eagle owl/Sweden/V618/06 | K | D | S | P | T | T | N | R | G | | A | K | | I | S | A | Q | G | N | PQGERRRKKRGLF | Y | | I | F | N | M | V | | R | N | D | L | V | A | S | G |  |
| A/Smew/Sweden/V820/06 | K | D | S | P | T | T | N | R | G | | A | K | | I | S | A | Q | G | N | PQGERRRKKRGLF | Y | | I | F | N | M | V | | R | N | D | L | V | A | S | G |  |
| A/Mute Swan/Sweden/V827/06 | K | D | S | P | T | T | N | R | G | | A | K | | I | S | A | Q | G | N | PQGERRRKKRGLF | Y | | I | F | N | M | V | | R | N | D | L | V | A | S | G |  |
| A/Mink/Sweden/V907/06 | K | D | S | P | T | T | N | R | G | | A | K | | I | S | A | Q | G | N | PQGERRRKKRGLF | Y | | I | F | N | M | V | | R | N | D | L | V | A | S | G |  |
| A/Canada goose/Sweden/V978/06 | K | D | S | P | T | T | N | R | G | | A | K | | I | S | A | Q | G | N | PQGERRRKKRGLF | Y | | I | F | N | M | V | | R | N | D | L | V | A | S | G |  |
| A/Tufted duck/Sweden/V998/06 | K | D | S | P | T | T | N | R | G | | A | K | | I | S | A | Q | G | N | PQGERRRKKRGLF | Y | | I | F | N | M | V | | R | N | D | L | V | A | S | G |  |
| A/Herring Gull/Sweden/V1116/06 | E | D | S | P | K | I | K | K | S | | T | K | | I | S | A | Q | G | D | PQGERRRKKRGLF | Y | | V | S | N | T | I | | C | S | G | L | V | A | S | G |  |
| A/Tufted duck/Sweden/V1027/06 | K | D | S | P | T | T | N | R | G | | A | K | | I | S | A | Q | G | N | PQGERRRKKRGLF | Y | | I | F | N | M | V | | R | N | D | L | V | A | S | G |  |
| A/Eagle owl/Sweden/V1218/06 | K | D | S | P | T | T | N | R | G | | A | K | | I | S | A | Q | G | N | PQGERRRKKRGLF | Y | | I | F | N | M | V | | R | N | D | L | V | A | S | G |  |
|  |  |  |  |  |  |  |  |  |  | |  |  | |  |  |  |  |  |  |  | |  | | | | | | | | | | | | | | | |

Rows representing subclade 2.2.1. viruses are shaded.
